# In vivo biocompatibility of two PEG/PAA interpenetrating polymer networks as corneal inlays following deep stromal pocket implantation

**DOI:** 10.1007/s10856-012-4848-3

**Published:** 2013-01-26

**Authors:** Xiao Wei Tan, Laura Hartman, Kim Peng Tan, Rebekah Poh, David Myung, Luo Luo Zheng, Dale Waters, Jaan Noolandi, Roger W. Beuerman, Curtis W. Frank, Christopher N. Ta, Donald TH Tan, Jodhbir S. Mehta

**Affiliations:** 1 Tissue Engineering and Stem Cell Research Group, Singapore Eye Research Institute, 11 Third Hospital Avenue, Singapore, Singapore; 2 Department of Chemical Engineering, Stanford University, Stanford, CA USA; 3 Department of Bioengineering, Stanford University, Stanford, CA USA; 4 Department of Ophthalmology, Stanford University, School of Medicine, Stanford, CA USA; 5 Department of Ophthalmology, Yong Loo Lin School of Medicine, National University of Singapore, Singapore, Singapore; 6 Department of Clinical Sciences, Duke-NUS Graduate Medical School, National University of Singapore, Singapore, Singapore

## Abstract

This study compared the effects of implanting two interpenetrating polymer networks (IPNs) into rabbit corneas. The first (Implant 1) was based on PEG-diacrylate, the second (Implant 2) was based on PEG-diacrylamide. There were inserted into deep stromal pockets created using a manual surgical technique for either 3 or 6 months. The implanted corneas were compared with normal and sham-operated corneas through slit lamp observation, anterior segment optical coherence tomography, in vivo confocal scanning and histological examination. Corneas with Implant 1 (based on PEG-diacrylate) developed diffuse haze, ulcers and opacities within 3 months, while corneas with Implant 2 (based on PEG-diacrylamide) remained clear at 6 months. They also exhibited normal numbers of epithelial cell layers, without any immune cell infiltration, inflammation, oedema or neovascularisation at post-operative 6 month. Morphological studies showed transient epithelial layer thinning over the hydrogel inserted area and elevated keratocyte activity at 3 months; however, the epithelium thickness and keratocyte morphology were improved at 6 months. Implant 2 exhibited superior in vivo biocompatibility and higher optical clarity than Implant 1. PEG-diacrylamide-based IPN hydrogel is therefore a potential candidate for corneal inlays to correct refractive error.

## Introduction

According to a recent review, 95.4 % patients worldwide undergoing LASIK were satisfied with their outcomes [1], making LASIK one of the most successful elective procedures performed. The remaining 4.6 % of these patients (approximately 750,000 people) were however dissatisfied with the procedure. Causes of dissatisfaction were associated with corneal haze, diffuse lamellar keratitis, flap-related problems, epithelial ingrowth, corneal ectasia and dry eye. While improvements in laser technologies that include the use of eye trackers, smoother corneal ablations and customised ablation profiles may reduce some of these problems, all laser-based procedures run the inherent intrinsic risk of causing irreversible ablation to the cornea. One of the most serious complications of laser-based procedures is LASIK-induced ectasia [2]. The identification of patients at risk of ectasia is a major difficulty for refractive surgeons. Although many risk factors for post-surgical ectasia have been identified, they do not indicate causation, and even patients with no known risk factors may develop ectasia [2–4]. It is this unpredictability that has pushed research into developing non-ablative methods for correcting refractive errors. A relatively new technique is the implantation of diffractive or refractive multifocal intracorneal lenses to alter the curvature of the cornea or the refractive index of the material itself [5].

A corneal inlay procedure involves the placement of a synthetic lens into the corneal stroma to correct the refractive error [5]. The advantage of an inlay procedure is that it adds a degree of reversibility to the refractive correction since no tissue is removed permanently. Efforts to develop corneal inlays are not new. In 1949, Barraquer [6] used a synthetic inlay made of impermeable flint glass and plexiglass to correct refractive errors. Other researchers have worked with different materials, including polysulphone [7], pHEMA [8], Permalens [9], Collagen IV [10], Collagen VI [11], Nutrapore (Hydrogel) [12], and perfluoropolyether [13]. The problem with most biological inlay materials is that are susceptible to biodegradation by host proteases, and they do not have enough permeability to support a healthy corneal epithelium [11]. While some synthetic polymers are more stable than others in a stromal environment, they may lack the porosity to support optimal corneal nutrient flow. Complications related to this include lipid deposition, crystal formation, opacification, peripheral ulceration, fibrosis and vascularisation [14, 15]. The long list of complications has led researchers to develop novel biomaterials. In addition to biostability, the ideal polymer for a corneal inlay is chemically inert, transparent to light, permeable to nutrient flow, easy to handle, sterilisable, and possesses a modulus similar to corneal tissue [16, 17].

Interpenetrating polymer networks (IPN) are a unique group of compounds composed of two or more independently cross-linked polymers. They are typically produced by first synthesising one network, swelling it in a second aqueous monomer solution and polymerising the latter to form a water-swollen mesh of two different polymers [18]. The hydrophilicity and hydrophobicity of an individual IPN can be controlled by varying the selection of the original monomers. Likewise, two polymers are often combined synergistically so that the beneficial properties of both polymers outweigh the drawbacks of an individual polymer. We have been developing an IPN consisting of a neutral cross-linked polymer (end-linked PEG [poly(ethylene glycol)] macromonomer), of defined molecular weight, as the first network and a charged ionised loosely cross-linked polymer (PAA [poly(acrylic acid)]) as the second [19]. PEG is a biocompatible polymer used extensively as a biomaterial in medicine. It is soluble in aqueous solutions and can be easily modified on exposure to UV light to form cross-linked hydrogels of high water content [20]. PAA is an anionic polyelectrolyte that is also used in biomedical devices for its absorbent capacity. The combination of PEG and PAA in an IPN forms an optically transparent, homogenous hydrogel with good mechanical properties [21] and a glucose diffusion coefficient similar to that of human cornea [22].

The aim of this in vivo study was to assess the biocompatibility and optical clarity of two IPN materials consisting of PEG/PAA double networks when used as corneal inlay in a rabbit deep corneal stromal pocket implantation model. PEG-Diacrylate was the monomer used in the creation of one of the cross-linked polymers of Implant 1 and PEG-Diacrylamide was the monomer used in the creation of one of the cross-linked polymers of Implant 2. The second meshworks in Implant 1 and Implant 2 are the same, which was PAA. Details about the chemical composition of the two IPNs hydrogels have been previously described [21, 22]. The advantages of an IPN over a single network are greater mechanical stability and higher water content.

## Materials and methods

The animal protocol of the study adhered to the Association for Research in Vision and Ophthalmology Statement for Use of Animals in Ophthalmic Vision and Research, and was approved by the Institutional Animal Care and Use Committee at Singapore Eye Research Institute, Singapore.

### Inlay material

As per standard procedure for the formation of an IPN, we used 50 % weight of PEG-macromonomer (MW: 4600 Da) in water and a photoinitiator (Irgacure 2959 5 % v/v). This produced a single PEG network. The single networks were subsequently soaked in a solution of acrylic acid monomer, a cross linker and photoinitiator. The acrylic monomers align along the backbone of the first network and thereby form an ordered PAA network within the PEG network. The IPN was then washed in phosphate buffer saline (PBS) until the pH and salt concentrations were equilibrated. To incorporate the second network, the single network hydrogel was removed from the mold and immersed overnight in an aqueous acrylic acid monomer solution (50 v/v %) containing photoinitiator stock solution (0.14 mmol per 1 ml acrylic acid) and triethyleneglycol dimethacrylate/tetra-ethyleneglycol diallylether as a crosslinking agent (0.04 mmol per 1 mL acrylic acid) [23]. The difference between the two IPNs used in this study was the choice of PEG based monomer. Implant 1 used PEG-diacrylate as its first network and Implant 2 used PEG-diacrylamide as its first network. The materials have diffusion coefficients for oxygen (30–60 Barrer) and glucose permeability (2.5 × 10^−6^ cm^2^/s). All materials were immersed in PBS and sterilized with ultraviolet UV for 30 min before implantation. The 30–40 μm thick implants were trephined with a dermatological trephine to 4 mm in diameter before implantation.

### Surgery

Twenty-four New Zealand White rabbits, aged 1–2 months and weighing 2–2.5 kg were used in the study. Animals were anaesthetised using an intramuscular injection of ketamine hydrochloride (35 mg/kg; Parnell Laboratories, Alexandria, Australia) and xylazine hydrochloride (5 mg/kg; Troy Laboratories, Smithfield, Australia). One eye of each rabbit was chosen at random for surgery. The contralateral eye of the implanted rabbit served as the control. Two drops of tetracaine (Chauvin Pharmaceuticals, Kingston Upon Thames, UK) were applied to the selected eye. Following anaesthesia, the rabbit eye to be implanted was prolapsed anteriorly to improve surgical access. An anterior lamellar dissection was performed using a crescent blade. The centre of the cornea was first marked with a keratoplasty trephine (Solan; Xomed Surgical Products Inc. Jacksonville, FL, USA). A guarded diamond knife (Storz, Bausch and Lomb, USA) was set to 75 % corneal depth and a 3 mm circumferential incision was made to enable the formation of a pocket incision and hence preservation of the corneal nerves [24]. A depth of 75 % has been previously shown to be the optimal position for an implant for nutritional flow (glucose and oxygen) in cornea stroma [25]. A deep lamellar dissection was then made, down to the posterior third of the cornea. A closed dissection technique was used by maintaining the anterior lamellar flap in its original position close to the stromal bed and the crescent knife (Sharpoint, Angiotech, Vancouver, Canada), was sandwiched between the 2 layers. With forceps gently holding the anterior flap in place, the knife was gradually advanced perpendicular to the cornea curvature in a gentle sweeping motion, thus splitting the cornea along a single lamellar plane. Sandwiching the blade between the stromal layers (and ensuring no tissue distortion occurs) resulted in an even dissection, increasing the likelihood of the dissection staying in the same tissue plane throughout. A 4-mm diameter implant was then folded and inserted into the pocket. Pocket incisions have been previously shown to maintain the nervous innervation of the cornea compared to a conventional LASIK like lamellar flap [25]. The implantation was done sequentially starting with Implant 1. Following insertion, the implant was unfolded using a Paton spatula (Storz Instrument, Bausch & Lomb, USA) with a gentle sweeping motion. The 3 mm incision was then sutured with interrupted 10/0 nylon sutures. All rabbits received the following topical medications post-implantation: neomycin-polymyxin B-dexamethasone ointment (Maxitrol; Alcon Laboratory, Inc., Fort Worth, TX, USA) four times per day, Pred Forte (Allergan; Irvine, California, USA) four times per day, atropine 1 % (Allergan; Irvine, California, USA) twice daily and a lubricating viscous gel (Vidisic Gel; Mann Pharma, Berlin, Germany) once a day for 1 week. Sutures were removed selectively in all rabbits at either 2 or 3 weeks post-implantation.

### Clinical examination

Slit lamp biomicroscopy was used to examine the rabbit corneas until the animals were sacrificed. The evaluation included assessment of the corneal incision, conjunctival vasculature, appearance of the cornea anterior and posterior to the inlay, position of the inlay and the optical clarity. Optical clarity of the inlays was assessed using biomicroscopy 30 min after a single application of a topical mydriatic eye drop (0.5 % tropicamide, Mydrin P; Santen, Osaka, Japan), and back-scattered light was graded using a previously described scale [16]. Animals were sacrificed at predetermined times of 3 and 6 months after surgery with an intravenous injection of sodium pentobarbital (100 mg/kg, Nembutal; Dainabot, Osaka, Japan). The rabbit eyes were then enucleated and processed for histology.

### Anterior segment optical coherence tomograph (ASOCT)

ASOCT imaging (Visante; Carl Zeiss Meditec, Dublin, CA) was performed in a room with standard lighting. The rabbits were held close to the chin rest so that the eyes were within the focal range of the machine. One scan (anterior segment scan) centred over the pupil was taken along the horizontal axis (between 0° and 180°). The scan direction was aligned until a full corneal reflex was achieved (indicated by an interference flare on the axis of the anterior chamber). The fixation angle was adjusted to obtain a horizontal image. The best quality image of each rabbit eye (as judged by visibility of the sclera spur and maximum interference flare) was selected.

### Corneal topography

Corneal topography was studied using a hand-held videokeratographer (Oculus, Lynnwood, WA). The device was aimed at the centre of the cornea to ensure centration of the smallest Placido mire ring (the instrument measurement axis) for all eyes. The Medmont E300 W has a 32-ring Placido cup target and can provide corneal coverage over areas from 0.25 to 11 mm in diameter. In this instrument, manual alignment and automatically initiated image capture was combined with storage of selected images. Each image was automatically analysed for centering, focus and stability and was rated to yield a percentage figure for quality, after correction for detected defocus and alignment errors. Each image analysed was edited using a scale step size of 0.1 D to locate the steepest area. Cursor movement over the steepest area allowed the steepest point curvature to be determined.

### In vivo confocal scanning

In vivo confocal microscopy was performed with the Heidelberg Retina Tomograph II Rostock Corneal Module (RCM; Heidelberg Engineering GmBH, Dossenheim, Germany). Examinations were performed preoperatively and every month post-implantation. The RCM is a class 1 laser system, utilises a 670 nm red wavelength diode laser source and does not pose any ocular safety hazard. However, to guarantee the safety of the operator and the rabbits, the maximum period of a single exposure was set to 30 min. A 60× objective water immersion lens with a numerical aperture of 0.9 (Olympus, Tokyo, Japan) and a working distance, relative to the applanation cap, of 0.0–3.0 mm was used. The dimensions of each image produced using this lens are 400 × 400 μm, and the manufacturers quote transverse resolution and optical section thickness as 2 and 4 μm, respectively.

All rabbits were anaesthetised with a drop of tetracaine (Chauvin Pharmaceuticals, Kingston Upon Thames, UK). Viscotears (Carbomer 980, 0.2 %; Novartis, North Ryde, NSW, Australia) was used as a coupling agent between the applanation lens cap and the cornea. During the examination, all rabbits were held stationary, and their lids were held apart to enable examination of the central cornea. The full thickness of the central cornea (within the central 2 mm diameter) was scanned using the device’s video mode. The video mode captures sections with 2 μm interval, up to 20 μm from the initial desired depth. Three frames per location with the clearest images were used for comparison of implanted, sham and control eyes. Any blurred or non-tangential images were excluded. Images were taken to include the corneal stroma, anterior and posterior to the implant.

### Histology (light and electron microscopy)

Eyes from all animals after euthanasia were fixed immediately in ice-cold 2.0 % glutaraldehyde, 2 % paraformaldehyde and 0.1 M sodium cacodylate buffer, pH 7.4, and subjected to histological analysis. Tissue sections were cut and stained with hematoxylin and eosin (Sigma-Aldrich, USA). For transmission electronic microscopy (TEM) imaging, the tissues were trimmed into smaller pieces. These samples were post-fixed in 1 % osmium tetra-oxide (Electron Microscopy Sciences, Washington, USA) and after extensive rinsing with sodium cacodylate buffer, dehydrated in a graded series of ethanol and embedded in Araldite (Electron Microscopy Sciences, USA). All semi-thin sections of 0.5–1 μm thickness were cut with Reichert-Jung Ultracut E Ultramicrotome (C. Reichert Optische Werke AG, Vienna, Austria), counter-stained with toluidine blue/basic fuchsin stain and examined using an Axioplan, Zeiss Light Microscope (Carl Zeiss, Germany). All ultra-thin sections of 60–80 nm were collected on copper grids, doubled-stained with uranyl acetate and lead citrate for 20 min each, then viewed and photographed on a Philips EM 208 S Transmission Electron Microscope (FEI Electron Optics BV, Eindoven, The Netherlands) at 100 kV. The epithelial-stroma interphase region and stroma near the polymer insertion area were chosen for imaging.

## Results

### Clinical outcomes

Of the 24 rabbits that underwent surgery, nine rabbit eyes received Implant 1 (PEG-diacrylate-based IPN, *n* = 9) and 13 eyes received implant 2 (PEG-diacrylamide-based IPN, *n* = 13). There were four sham control eyes from two rabbits, where the surgery was performed without implantation. One of the rabbits with Implant 1 and two rabbits with Implant 2 developed microbial keratitis within the first week of implantation and were excluded from the study. A total of 8 rabbits had Implant 1 and 11 rabbits had Implant 2 successfully implanted. Two rabbits in the Implant 1 group developed progressive vascularisation starting from 5 to 7 weeks and hence were terminated earlier than originally planned. Two rabbits with Implant 1 developed epithelial defects and hence were both terminated at 2 months. A third rabbit with Implant 1 developed an epithelial defect at 3 months and hence was terminated at its original predetermined time point. Three rabbits with Implant 1 reached the 3 month time point but were then sacrificed. All had significant clinical haze over the implant. One of the rabbits with Implant 2 developed a focal area of superficial vascularisation and hence was terminated at 7 weeks post-operation. The remaining 10 rabbits with Implant 2 were maintained to the original predetermined time points without any problems. Implant 2 inlays were better tolerated over the 3 months period of study than Implant 1. They also all retained good biocompatibility over the 6 months period of study.

### Slit lamp

**Fig. 1:**
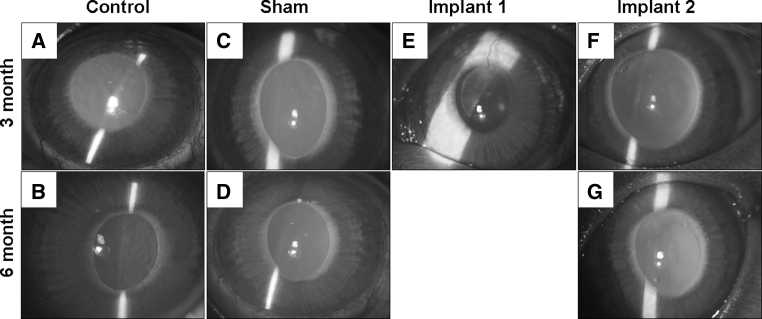
Representative slit lamp micrographs of the rabbit corneas. **a**, **b** Normal corneas at 3 or 6 month time point. **c**, **d** Sham-operated corneas at 3 or 6 month time point. **e** Cornea with Implant 1 at 3 month. **f**, **g** Corneas with Implant 2 at 3 month or 6 months

**Fig. 2:**
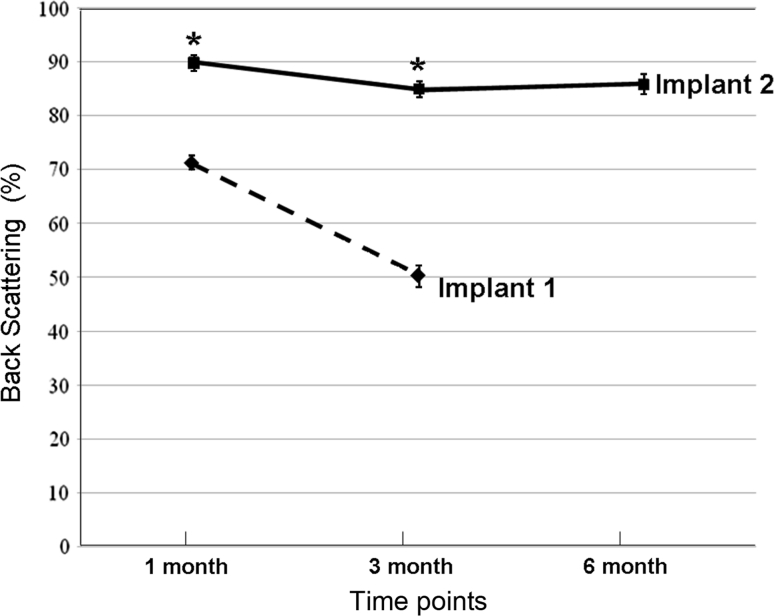
Optical clarity of the corneal inlays assessed by percentage back-scattering of light using a slit lamp microscopy and a grading scale. * *P* < 0.05 between Implant 1 and Implant 2. *n* = 4 for Implant 1, *n* = 10 for Implant 2 at post-operative 3 month or 6 month time point

Slit lamp examination showed that all control and sham-operated corneas were haze free at both post-operative 3 and 6 month (Fig. 1a, b and c, d). A diffuse haze and an ulcer were observed in one cornea with Implant 1 at post-operative 3 month (Fig. 1e) while all corneas with Implant 2 were clear at both post-operative 3 month and 6 month (Fig. 1f, g). In the 3 corneas with Implant 1 that survived for 3 months, polymer transparency decreased from 72 to 50 %. In contrast, the polymer transparency of Implant 2 was maintained around 85 % between the 3 month (*n* = 10) and 6 month (*n* = 5) time points (Fig. 2, *P* = 0.01).

### ASOCT and corneal topography

**Fig. 3:**
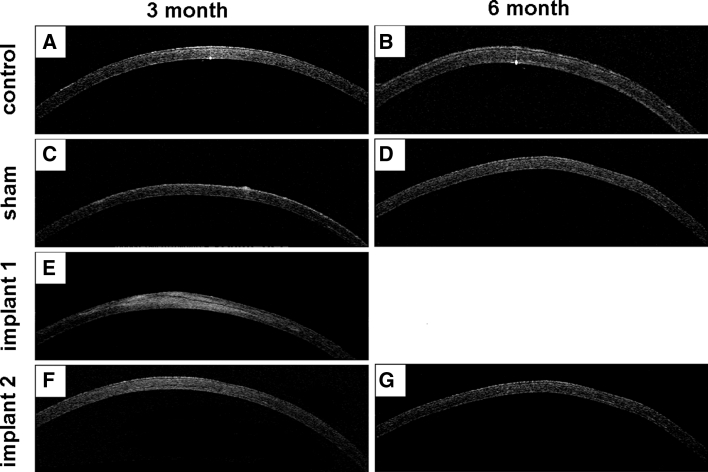
Representative cross sectional visualisation of rabbit corneas using ASOCT. **a**, **b** Normal corneas at 3 or 6 month time point. **c**, **d** Sham-operated corneas at 3 or 6 month time point. **e** Cornea with Implant 1 at 3 month. **f**, **g** Corneas with Implant 2 at 3 or 6 months

On ASOCT examination, the corneal inlay appeared as a dark line in the scanned image, indicating the insertion position of the implant. No abnormalities were noted in corneal thickness for normal control and sham-operated eyes (Fig. 3a, b and c, d). Increases in corneal thickness, corneal stromal light reflection and corneal surface curvature abnormality were observed in the OCT images of corneas with Implant 1 (Fig. 3e) but not in those of corneas with Implant 2 (Fig. 3f, g).

**Fig. 4:**
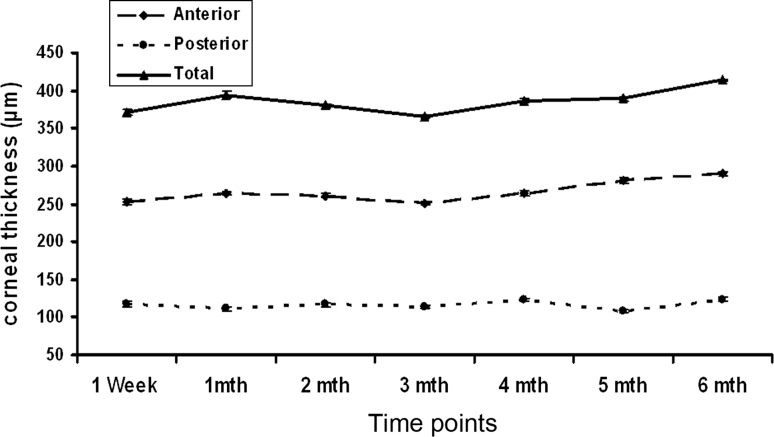
Total corneal thickness, anterior stromal thickness and posterior stromal thickness of corneas with Implant 2 assessed with ASOCT. *n* = 11 for post-operative 1 week group, *n* = 10 for post-operative 3 month group, *n* = 5 for post-operative 6 month group

Total corneal thickness of Implant 2 at post-operative 1 month was very close to pre-operative readings [394 ± 5 μm (*n* = 11) and 390 ± 2 μm (*n* = 13) respectively]. At post-operative 3 month time point, total corneal thickness decreased to 365 ± 2 μm (*n* = 10) and gradually recovered to 414 ± 7 μm (*n* = 5) at post-operative 6 month. Anterior stromal thickness mimicked the fluctuating trend of total corneal thickness while posterior stromal thickness showed no significant change (Fig. 4).

**Fig. 5:**
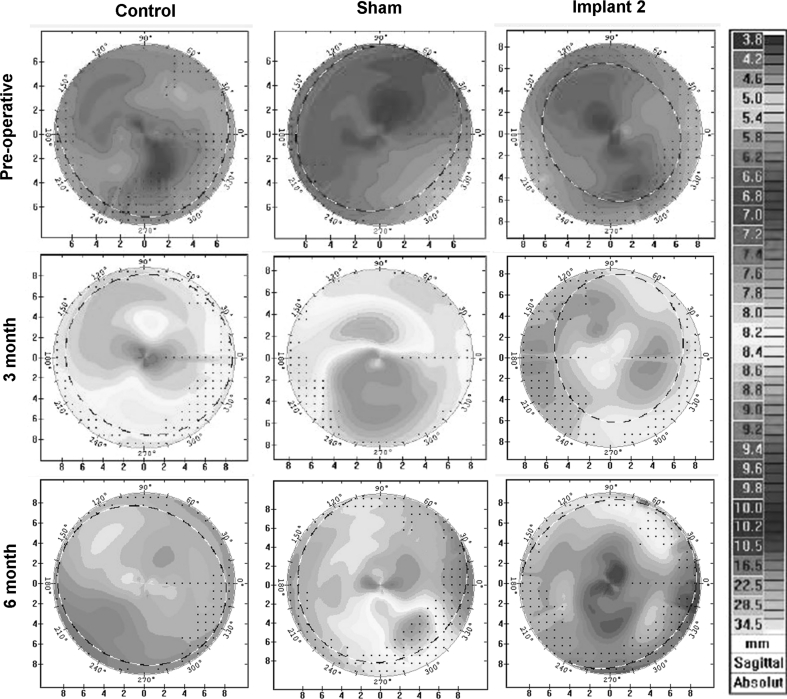
Confocal topography at pre-operative, post-operative 3 month and post-operative 6 month of control/sham operated cornea and cornea with Implant 2

**Fig. 6:**
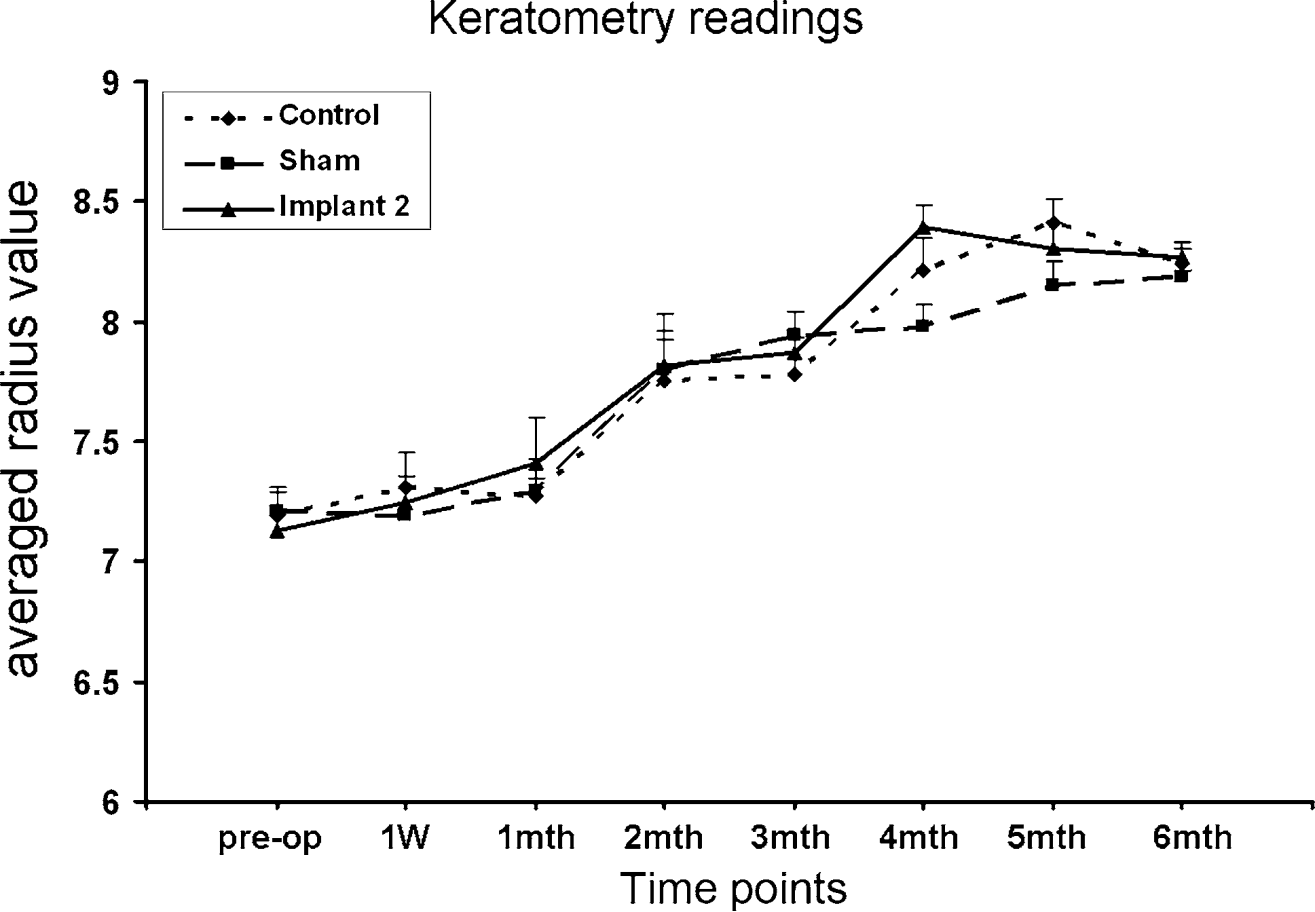
Keratometry readings of control cornea (*n* = 10), sham operated eye (*n* = 4) and cornea with Implant 2 (*n* = 10)

The corneal topography of rabbit cornea after Implant 2 insertions was analysed (Fig. 5). As the rabbits aged, we could see a more flattened zone (green zone of topography) was observed on all corneas of all the control, sham-operated and implanted eyes. The topography of implanted corneas was not obviously different from that of control/sham-operated corneas. Averaged radius values, determined from corneal topography, indicated more flattening of the corneas as the observation time increased. However, there was no significant difference in average radius among control corneas, sham-operated corneas and corneas with Implant 2 (Fig. 6). Before operation, the average radius values were 7.2 ± 0.09 mm for control eyes, 7.2 ± 0.1 for sham-operated eyes, 7.3 ± 0.14 mm for implanted eyes group (*P* > 0.05). At post-operative 3 month, the average radius values were 7.9 ± 0.1 mm for control eyes, 8.2 ± 0.2 mm for sham-operated eyes and 7.9 ± 0.2 for implanted eyes. At post-operative 6 month, the average radius values were 8.2 ± 0.7 mm for control eyes, 8.2 ± 0.03 mm for sham-operated eyes and 8.3 ± 0.1 for implanted eyes (*P* > 0.05).

### In vivo confocal microscopy

**Fig. 7:**
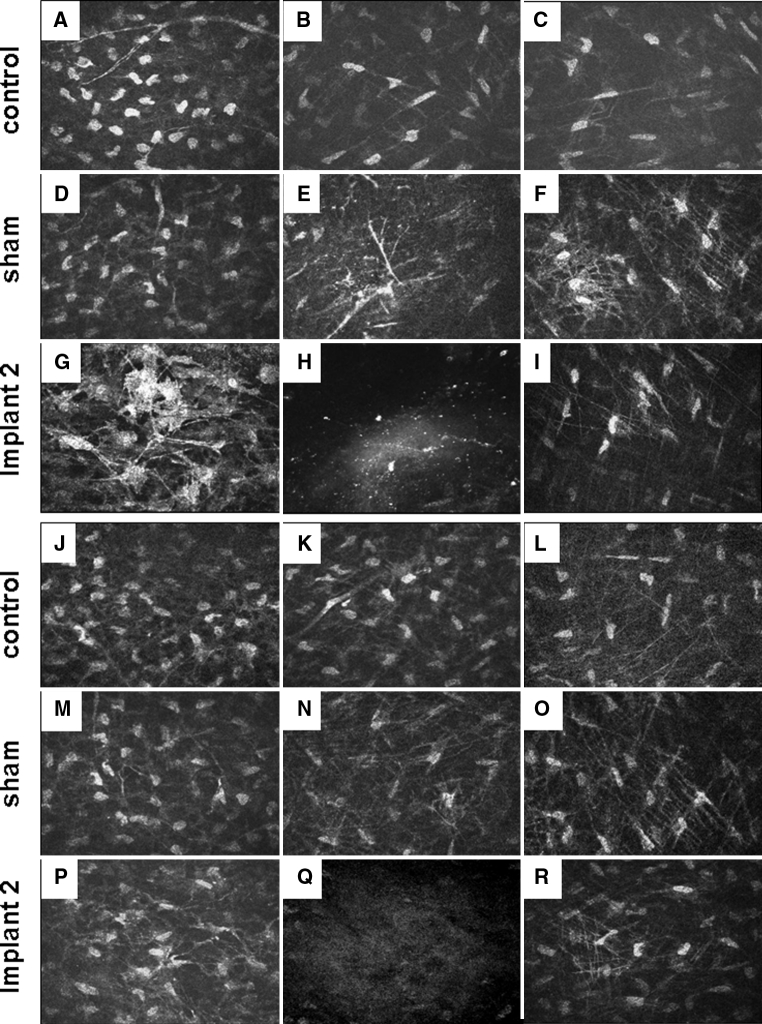
In vivo confocal scans of the rabbit cornea. **a**–**i**: post-operative 3 month; **j**–**r**: post-operative 6 month. *1st* and *4th row*: control rabbit; *2nd* and *5th row*: sham-operated cornea; *3rd* and *6th row*: cornea with Implant 2. *Left column* represents scans from the anterior stroma layer. *Middle column* represents the intrastroma layer and *right column* represents the posterior stroma layer. **e**, **n** represent the sham pocket area without any implant; **h**, **q** represent the scans of Implant 2 in the stromal layer at 3 or 6 month post-operative time point

Normal controls, sham-operated and corneas with Implant 2 were examined with in vivo confocal microscopy. At 3 months after implantation, a few highly reflective keratocytes could be seen in the stromal layer directly anterior to the corneal inlay (Fig. 7g). In normal and sham-operated corneas, the keratocytes only showed moderate light scattering (Fig. 7a, d). More intense and abundant reflective particles and nerve bundles were observed in the stromal layer of the operated corneas than in normal and sham-operated corneas. The implant itself was acellular and characterised by light-scattering particles (Fig. 7h). The keratocytes showed moderate light scattering in the posterior stromal layer of normal, sham-operated and implanted corneas and the intensities had no significant differences among the groups (Fig. 7c, f, i). Moderate immune cell infiltration and inflammation was observed by in vivo confocal microscopy at post-operational 3 months. At post-operative 6 month, the light reflectivity and keratocytes density in anterior and posterior stromal layer were similar among normal control, sham-operated and implanted corneas (Fig. 7j, m, p and l, o, r).

### Histology

**Fig. 8:**
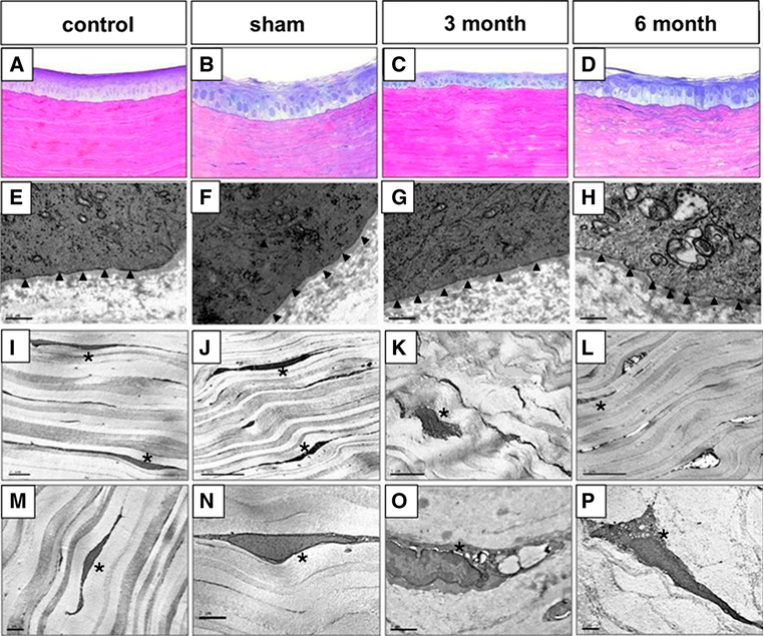
H&E staining and transmission electron micrograph sections of the central cornea epithelium. *1st row*: H&E staining, *2nd row*: transmission electron micrograph sections of the epithelial basement membrane area. Epithelium-stroma hemidesmosomes were seen along the basement membrane. *3rd* and *4th* *row*: low and high magnification transmission electron micrograph of the cornea stroma anterior to Implant 2. **a**, **e**, **i**, **m** represent Normal control rabbit; **b**, **f**, **j**, **n**: Sham operated corneas; **c**, **g**, **k**, **o**: Corneas with Implant 2 at post-operative 3 month; **d**, **h**, **l**, **p**: Corneas with Implant 2 at post-operative 6 month. Normal cornea and sham operative cornea were fixed at post-operative 3 month. *upright triangle* indicates the location of a hemidesmosomes and *asterisk* indicates the location of a keratocyte

Haematoxylin &Eosin sections showed that after 3 months the epithelia of the rabbit corneas were stratified but thinned compared with the central epithelium of a normal rabbit (Fig. 8c). The epithelial basal cells had become cuboidal rather than columnar and accounting for the thinning, there was a reduction in the number of epithelial cell layers. Before 6 months, the central epithelium had recovered to normal thickness, comparable with the control group.

Ultrastructural TEM of the central portion of each cornea in the study showed that hemidesmosomes, the cell–matrix junction responsible for the anchoring the epithelium to its underlying stroma, were present along the basement membrane with similar morphology and distribution to normal rabbit cornea (Fig. 8e–h). Keratocytes were observed at the epithelial-stroma interphase region in all corneas with/without implants (Fig. 8i–l). The keratocytes in the cornea stroma with the implant were more vacuolated than those in sham-operated and non-operated corneas (Fig. 8m–p). At 3 months, nuclear shrinkage and organelle swelling were observed, indicating keratocyte degeneration. At post-operative 6 months, the keratocytes showed less extensive vacuolation of the cytoplasm, organelle swelling and nucleus shrinkage compared to the post-operative 3-month levels. No neovascularisation and no oedema were observed in the vicinity of the inlays and in sham-operated corneas at both post-operative 3 and 6 months.

## Discussion

Two IPNs, based on PEG-diacrylate (Implant 1) and PEG-diacrylamide (Implant 2) were developed as possible artificial corneal inserts because of their high water content and mechanical stability [23]. To investigate and compare their biocompatibility with corneal stroma they were inserted into corneal stroma pockets created in rabbit eyes using a manual dissection surgical technique and examined over a period of 6 months. Corneas with Implant 1 showed diffuse haze, ulceration and opacification within 3 months whereas corneas with Implant 2 remained clear for up to 6 months. With Implant 2, corneal thickness and surface topography showed no significant changes from normal corneas at 6 months post-operation (Figs. 3, 4). Although edema and neovascularisation were not apparent in these corneas 3 months after surgery, moderate immune cell infiltration and inflammation was observed by in vivo confocal microscopy. Thinning of the epithelial layer over the hydrogel inserted area and keratocyte degeneration had also occurred by this time point but had begun to resolve by 6 months (Fig. 8). On the basis of a previous report by Hartmann et al. [23] that an IPN with the dimethacrylate-crosslinker (Implant 2) showed higher mechanical stability towards in vitro hydrolytic degradation than the IPN with the diallylether-crosslinker (Implant 1) [23] it is suggested that this may be the reason for the significant difference in performance of these IPN implants after insertion into rabbit corneas.

In vivo biocompatibility of corneal inlays depends on various factors including the material itself (permeable or non-permeable polymer), the surgical procedure, the test animals and post-operative conditions. Of these factors, the surgical procedure plays an important role in determining the tolerance of an inlay. In most mammals including humans, the majority of the stromal nerve fibers are located in the anterior third of the stroma [24]. The bundles enter the cornea at the periphery in a radial fashion parallel to the corneal surface exerting important trophic influences on the corneal epithelium and contributing to the maintenance of a healthy ocular surface [25]. Although LASIK is the commonest technique reported to aid inlay implantation [26, 27] there is complete transection of the sub-epithelial corneal nerves that may require 6 months to several years to heal [28] and the creation of a cornea flap causes permanent stromal damage because it never fully heals. For these reasons creating a deep corneal stromal pocket will cause less nerve damage than a flap and for this study we therefore adopted a surgical technique that allowed us to selectively evaluate the inlays in non-neurotrophic corneal tissue.

Corneal inlays have a long history of failed trials [7]. Early studies showed that the insertion of impermeable inlays in the cornea led to degeneration of the tissue anterior to the implant because of insufficient fluid flow and nutrition transport from the aqueous humour. Recently, Larrea et al. [29] using a computational model to simulate corneal nutrient transport reported that for oxygen transport, the minimum oxygen concentration was highest when the implant was placed at ¾ of the corneal thickness. Our implant materials have diffusion coefficients for oxygen and glucose similar to those of the human cornea (oxygen: 30 barrer and glucose: 2.5–3 × 10^6^cm^2^/s) [22] and were consistently implanted within deep corneal stromal pockets at 75 % corneal depth, as evidenced by ASOCT. These are therefore the likely reasons for the high success rate that we obtained using Implant 2. In previous smaller pilot series of animal studies using a microkeratome technique for implant placement, the clinical results were not as good as those achieved in this study [23].

The limitations of this study have been considered. We did not measure the endothelium and posterior curvature of the rabbit cornea because we know from patients who have undergone manual Deep Anterior Lamellar Keratoplasty (DALK), that there is minimal effect on the corneal endothelium and posterior curvatures. Further, because the IPN implants were not machine-finished but simply trephined, the blunt edges of the implant could have a detrimental effect on implant-tissue inflammatory cellular responses. For this reason the low level inflammatory response that we documented at 3 months in our manually cut implants might be expected to be reduced using machine cut implants. For the histological assessment of the implant-tissue interface, tissue processing would have been possible with the hydrogel in situ [22] but for operational ease and because little extra information would have been obtained, it was removed during tissue sectioning. Finally, the rabbits used in our study were young and their eyes still growing. With growth, the cornea curvature becomes more flattened and its thickness increased. This was observed in our corneal topography images (Figs. 5, 6) [30, 31] and with reference to the control eyes, compensated for when analysing the data. Given that the aim of the study was to compare implants inserted into the corneas of same age rabbits this manipulation could not affect the conclusions drawn.

Future work with Implant 2 will focus on refining techniques for surgical implantation and improving the physical quality of the implant itself. The development of the femtosecond laser to deliver precise intrastromal cuts offers a wide range of applications in corneal refractive surgery including corneal inlay insertion [32]. The laser can be delivered in a specified pattern and intrastromal depth that is set pre-operatively via computer software. To date there are a number of corneal inlays being evaluated in combination with femtosecond laser creation of pockets/flaps but most of the inlays currently available are for presbyopia [33–37]. Despite this, early clinical outcomes suggest that this combined procedure may ultimately be applicable to patients with myopia and hyperopia.

## Conclusion

In summary, by comparison with those based on PEG-diacrylate, the implantation of synthetic PEG/PAA IPN hydrogel inlays based on PEG-diacrylamide in a deep corneal stromal pocket with our manual surgery technique showed relatively good biocompatibility over a post-operative time of 6 months. Further studies are required to assess longer term biocompatibility and in vivo stability of corneal inlays based on PEG-diacrylamide.
